# Patterns of tobacco product single and co-use – Findings from the Population Assessment of Tobacco and Health Study (PATH) wave 7 data

**DOI:** 10.1371/journal.pone.0360653

**Published:** 2026-11-03

**Authors:** Shu Xu, Yelan Wang, Luchang Cui, Sarah Hofmeister, Raymond S. Niaura

**Affiliations:** New York University, New York, New York, United States of America; All India Institute of Medical Sciences, INDIA

## Abstract

**Objective:**

This study aimed to comprehensively assess and describe exclusive and combined use patterns across the full range of tobacco products, including emerging ones, to inform tobacco product surveillance.

**Methods:**

We analyzed Wave 7 data (collected between January 2022 and April 2023) from the Population Assessment of Tobacco and Health Study, including 10,453 youth and 29,645 adults. We reported the frequency and weighted prevalence of current use across up to eleven tobacco products and used UpSet plots to visualize intersections of current tobacco use. We also examined the intersections among current nicotine pouch users, as a secondary analysis. Among current tobacco users, we fit multinominal logistic regression models to assess the association between sociodemographic characteristics and tobacco use patterns, with the single-product use as the reference category.

**Results:**

Among youth, we identified 41 distinct patterns of single and combined use, representing 6.0% of the U.S. youth overall; single-, dual-, and poly-use accounted for 4.9%, 0.8%, and 0.3%, respectively. Compared with single-product users, dual-users were more likely to have lower annual household incomes, and poly-users were more likely to be male. Among adults, we identified 183 distinct patterns of single and combined use, representing 24.7% of US adults overall; single-, dual-, and poly-use accounted for 17.2%, 5.5%, and 2.0%, respectively. Compared with single-product users, dual- or poly-users were more likely to be older, male, non-Hispanic Black, and have low annual household income.

**Conclusions:**

Tobacco surveillance and research should routinely assess a broad range of products, including emerging products, and explicitly characterize co-use patterns. Our findings support policies and intervention strategies that address multiple-product use and target high-risk subgroups, rather than focusing on single-product use.

## Introduction

Tobacco use remains leading cause of preventable disease, mobility, and mortality in the United States (U.S.) [1]. Tobacco users may use a single product exclusively (single use), or concurrently use two (dual use) or three or more products (poly use). Distinguishing between exclusively single tobacco use and co-use patterns is essential for understanding the behavioral characteristics and health consequences associated with tobacco use. However, capturing the full spectrum of tobacco use patterns remain challenging because of availability of new and rapidly evolving tobacco products and the complexity of concurrent product use behaviors.

Accumulating evidence suggests that dual and poly-tobacco users differ from single-product users. Dual and poly-users may experience greater nicotine dependence [2], more positive attitudes toward tobacco [3,4], lower quit intent [5–10], and lower cessation rates [10], compared with exclusive users. The recent increasing prevalence of dual and poly-use is likely driven by the emergence and proliferation of newer products such as e-cigarettes [5–9], heat-not-burn products [11], and recently, nicotine pouches [12,13]. Concurrent use of multiple tobacco products is associated with elevated risk of adverse health outcomes. Compared with exclusive use of a single product, dual and poly-users of conventional and novel nicotine products (e-cigarettes, heated tobacco) may experience higher cumulative intake of nicotine and other harmful substances [14–16]. Studies indicate that dual users may exhibit biomarkers of nicotine and toxicant exposure that are at least as high as those of exclusive cigarette smokers and substantially higher than those of exclusive e-cigarette users [17]. Dual or poly-users have been shown to have similar or higher levels of serum cotinine and urinary total NNAL (4-[methylnitrosamino]-1-[3-pyridyl]-1-butanol, which play important roles in cancer) than single users [18], and this may depend on the intensity of cigarette use. However, it is often unclear which specific combinations of dual- or poly-tobacco use are associated with these observed behavioral or health outcomes.

Comprehensive surveillance of tobacco use is increasingly important given the rapid evolving tobacco product landscape. Although cigarette smoking has declined steadily over decades, use of newer nicotine products, especially e-cigarettes, has increased substantially [19]. However, current surveillance efforts often fail to capture the full complexity of tobacco use behaviors. Most studies focus on the prevalence of individual products and classify users without accounting for the concurrent use other tobacco products [20]. Even when dual or poly tobacco use is examined, studies frequently report broad categories of co-use without specifying the exact combination of products involved [15,21–23]. Different combinations of tobacco products may be associated with distinct health risks, behavioral profiles, and intervention needs [18]. Given the dynamic nature of tobacco use behaviors, as illustrated by recent market surveillance data showing substantial shifts in dual and poly tobacco use patterns between 2021 and 2024, a comprehensive understanding of current tobacco exclusive single and co-use patterns that capture the full spectrum of tobacco use patterns is required.

To our knowledge, no study has comprehensively assessed the full range of tobacco products used exclusively and in combination among U.S. youth and adults. Hence, in this study, we estimated current (past 30-day) use of tobacco products and used data visualization tools to examine patterns of single, dual, and poly-tobacco product use among U.S. youth and adults. The analysis characterizes contemporary use behaviors, identifies sociodemographic correlates of co-use, and potentially will inform efforts to reduce tobacco-related harms.

## Methods

### Data sources and study participants

This study uses data from the Population Assessment of Tobacco and Health (PATH) Study, a longitudinal and nationally representative cohort of non-institutionalized youth (aged 12–17 years) and adults (aged ≥ 18 years), including smokers and non-smokers. A primary goal of the PATH Study is to assess population use of tobacco products and their health consequences following the Food and Drug Administration’s authority to regulate tobacco products, granted by Congress in 2009 (see Hyland et al, 2017 [24], for details on study design). All individual-level data were deidentified for public use. For this analysis, data were accessed on September 26th, 2025. we restricted the sample to Wave 7 (January 2022 – April 2023): 10453 youth and 29645 adults. We excluded 197 youth (1.9%) and 135 adults (0.5%) with missing values on any tobacco use item, assuming this small proportion of missing values were ignorable [25].

### Measures

*Tobacco use type*. We used PATH-derived indicators of current use – defined as use on at least one day in the past 30 days – for each product (11 distinct products for youth: cigarettes, e-cigarettes, cigar [including traditional cigar, filtered cigar, and cigarillo], pipe, hookah, snus, smokeless tobacco, bidi, IQOS, nicotine pouches, and oral tobacco; ten distinct products for adults: cigarettes, e-cigarettes, cigar, pipe, hookah, snus, smokeless tobacco, IQOS, nicotine pouches, and oral tobacco; 1 = yes, 0 = no). Based on the number of endorsed products, tobacco use type was categorized as no use (0 = 0 products), single (1 = 1 product [reference]), dual (2 = 2 products), and poly use (3 = ≥ 3 products). *Covariates.* Participants reported their sociodemographic characteristics, including age group (in years; youth: 12–14 [reference group], 15–17; adults: 18–24 [reference group], 25–34, 35–44, 45–54, 55–64, ≥ 65), sex (male, female [reference group]), and race/ethnicity (Hispanic, Non-Hispanic [NH] White [reference group], NH Black, and Other). Annual household income (<$50,000, >= $50,000 [reference group], Don’t know) and highest education attainment (Less than high school, High school graduate or GED [reference group], Some college or associates degree, Bachelor’s degree, and Advanced degree) were reported by youth’s parents or adults. Detailed descriptions of the study variables are presented in S1 Table for youth and S2 Table for adults.

### Analytical plan

We used similar analytic approaches for youth and adults, as detailed below. First, we generated descriptive statistics for each sample, reporting frequencies and unweighted and weighted proportions for tobacco products and covariates. Second, we used UpSet plots to illustrate the intersection of binary current tobacco use. These plots display: (1) a bar chart of intersection sizes (y-axis = frequency), and (2) a matrix of dots/lines indicating which products are included in each intersection (filled dot = included; empty = excluded), with side bars showing the sample size of each individual product used. Intersections were ordered by unweighted size, and weighted proportions were displayed below the corresponding bar. Given the rapid increase in nicotine pouch use among both youth and adults, we also conducted a secondary analysis to examine use patterns among current nicotine pouch users. Lastly, among current tobacco users, we constructed weighted multinominal logistic regression models to examine the association between covariates and tobacco use types, using single-product use as the reference category. Weighted estimates were computed using PATH Wave 7 cross-sectional sampling weights. Standard errors were estimated using balanced repeated replication with replication weights and Fay’s adjustment factor of 0.3 [26].

*Software.* We conducted descriptive analyses and multinominal logistic regression in SAS 9.4 [27], accounting for the complex four-stage sample design. We used the R UpSetR package to visualize intersections of tobacco use behaviors and their corresponding frequencies and proportions [28].

## Results

Descriptive statistics for the youth and adult samples are presented in Table 1. Among youth, about half were 12–14 years old (51.0%), male (51.2%), and NH White (48.9%). Close to half had a parent with a Bachelor’s degree or higher (47.7%). In addition, 63.8% were from families with an annual household income of $50,000 or higher.

**Table 1:** Characteristics of current tobacco user among youth and adult samples.

Variable	n	Unweighted %	Weighted %	Variable	n	Unweighted %	Weighted %
Youth	10453			**Adult**	29645		
Age (in years)							
12-14	4775	45.7	51.0	18-24	10,267	34.6	11.7
15-17	5678	54.3	49.0	25-34	6,922	23.4	17.4
				35-44	3,536	11.9	16.9
				45-54	2,835	9.6	15.7
				55-64	2,968	10.0	16.9
				65 +	3,116	10.5	21.4
				(missing)^a^	1		
Sex							
Female	4980	47.6	48.8	Female	15,633	52.7	51.5
Male	5473	52.4	51.2	Male	14,012	47.3	48.5
Race/Ethnicity							
Hispanic	3,103	29.9	25.6	Hispanic	6,790	22.9	16.9
NH White	5,015	48.2	48.9	NH White	15,600	52.6	61.2
NH Black	1,192	11.5	12.7	NH Black	4,747	16.0	11.3
Other	1,085	10.4	12.8	Other	2,508	8.5	10.6
(missing) ^a^	58						
Education							
< High School	905	8.7	8.0	< High School	2,949	10.0	9.2
High School/GED	1,888	18.2	17.2	High School/GED	1,451	4.9	4.7
Some College/Associates Degree	2,888	27.8	27.2	College/Associates Degree	7,242	24.5	23.3
Bachelor’s Degree	2,215	21.4	22.4	Bachelor’s Degree	10,271	34.8	29.7
Advanced Degree	2,484	23.9	25.3	Advanced Degree	7,623	25.8	33.1
(missing) ^a^	73			(missing) ^a^	109		
Annual Household Income							
>=$50,000	6,479	62.0	63.8	>=$50,000	13,848	46.7	53.6
< $50,000	3,728	35.6	33.8	< $50,000	14,713	49.6	42.9
Don’t Know	246	2.4	2.4	Don’t Know	1,084	3.7	3.5
Tobacco Current Use ^b^							
Cigarette	123	1.2	1.1	Cigarette	6,289	21.2	15.6
E-cigarette	617	5.9	5.3	E-cigarette	4,714	15.9	8.9
Cigar	45	0.4	0.4	Cigar	2,141	7.2	4.8
Pipe	11	0.1	0.1	Pipe	217	0.7	0.5
Hookah	6	0.1	0	Hookah	655	2.2	1.3
Snus	12	0.1	0.1	Snus	242	0.8	0.6
Smokeless Tobacco	11	0.1	0.1	Smokeless Tobacco	673	2.3	2.1
IQOS	2	0	0	IQOS	32	0.1	0.1
Nicotine Pouch	28	0.3	0.2	Nicotine Pouch	370	1.2	0.8
Oral Tobacco	17	0.2	0.2	Oral Tobacco	192	0.6	0.5
Bidi	4	0	0				

^a^The number of participants with missing data is reported for each covariate. Given the small amount of missingness, these observations were considered ignorable and were not included in the calculation of unweighted or weighted proportions.

^b^*n* represents the number of participants who reported current use of each tobacco product.

Among youth who currently use tobacco, we identified 41 distinct patterns of single and combined use across 11 tobacco products, representing 6.0% of the US youth overall; single, dual, and poly-use accounted for 4.9%, 0.8%, and 0.3%, respectively. As shown in Fig 1, the most commonly used products were e-cigarettes, cigarettes, cigars, nicotine pouches, unspecified other tobacco, and snus (overall use). Among single users, the leading products were e-cigarettes (4.3%), cigarettes (0.3%), cigars (0.1%), other oral tobacco (0.1%), and pipe tobacco (0.1%), with nicotine pouches as the sixth most common exclusively used product (0.03%). The most frequently reported dual-use patterns were co-use of cigarettes and e-cigarettes (0.6%), e-cigarettes and cigars (0.1%), and e-cigarettes and nicotine pouches (0.1%). Among poly-users, the most frequent reported patterns were cigarette-e-cigarette-cigar (0.1%), and other patterns (e.g., e-cigarette-nicotine pouch-snus, cigarette-e-cigarette-nicotine pouch, e-cigarette-cigar-nicotine pouch) account for less than 0.1%. Among the 28 nicotine pouch users (S1 Fig) in the secondary analysis, 4 (14.5%) exclusively used nicotine pouches, 8 (29.4%) co-used nicotine and e-cigarettes, and the rest (56.9%) used three or more products. Lastly, multinominal logistic regression results (Table 2) indicate that, among current tobacco users, boys had higher odds of poly-product use versus single-product use (adjusted odds ratio [AOR] = 5.65, 95% confidence interval [CI] = 2.38, 13.40); lower annual household income was associated with higher odds of dual-product use (AOR = 1.73; 95% CI = 1.02, 2.94). Tobacco use type was not associated with age or race/ethnicity. Small subgroup sample sizes for parents’ highest education level limited our ability to test their associations with tobacco use types.

**Fig 1:**
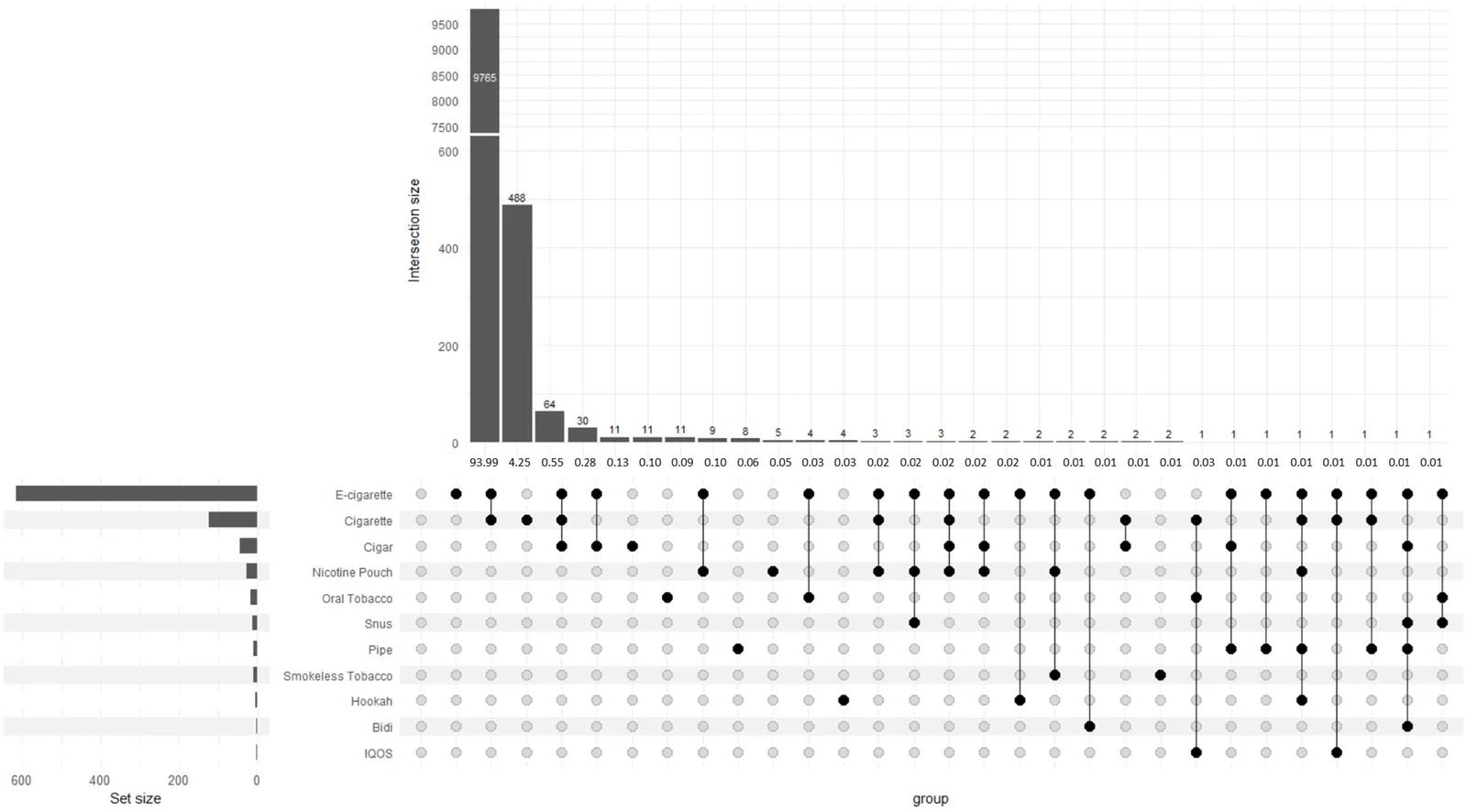
Upset plot of current tobacco use among youth (n = 10453). This plot, illustrating the intersection of binary current tobacco use, display: (1) a bar chart of intersection sizes (y-axis = frequency), and (2) a matrix of dots/lines indicating which products are included in each intersection (filled dot = included; empty = excluded), with side bars showing the sample size of each individual product used. Intersections were ordered by unweighted size, and weighted proportions were displayed below the corresponding bar. This figure displays up to 30 intersection patterns.

**Table 2:** Association between tobacco use type and covariates.

		AOR	95% CI	AOR	95% CI
		Dual- vs. Single Users		Poly- vs. Single Users	
**Youth**					
	Age (in years)				
	12–14 (ref)				
	15-17	1.40	[0.74, 2.65]	1.19	[0.28, 5.01]
	Sex				
	Female (ref)				
	Male	1.55	[0.91, 2.63]	**5.65**	**[2.38, 13.4]**
	Race/Ethnicity				
	Hispanic	0.67	[0.35, 1.29]	0.35	[0.07, 1.78]
	NH White (ref)				
	NH Black	0.64	[0.23, 1.78]	0.18	[0.02, 1.96]
	NH Other	0.84	[0.33, 2.10]	0.69	[0.14, 3.51]
	Annual Household Income				
	>=$50,000 (ref)				
	< $50,000	**1.73**	**[1.02, 2.94]**	1.16	[0.49, 2.75]
	Don’t Know	0.98	[0.09, 10.98]	3.74	[0.38, 36.80]
**Adult**					
	Age (in years)				
	18–24 (ref)				
	25-34	1.17	[0.99, 1.37]	1.00	[0.80, 1.27]
	35-44	**0.82**	**[0.68, 0.97]**	**0.63**	**[0.47, 0.86]**
	45-54	**0.68**	**[0.56, 0.83]**	**0.44**	**[0.31, 0.61]**
	55-64	**0.39**	**[0.32, 0.48]**	**0.21**	**[0.14, 0.32]**
	65 +	**0.31**	**[0.24, 0.40]**	**0.13**	**[0.07, 0.26]**
	Sex				
	Female (ref)				
	Male	**1.16**	**[1.02, 1.31]**	**1.87**	**[1.55, 2.27]**
	Race/Ethnicity				
	Hispanic	0.94	[0.80, 1.10]	0.95	[0.75, 1.22]
	NH White (ref)				
	NH Black	**1.23**	**[1.06, 1.44]**	**1.48**	**[1.13, 1.94]**
	NH Other	1.08	[0.83, 1.40]	1.00	[0.67, 1.47]
	Annual Household Income				
	>=$50,000 (ref)				
	< $50,000	**1.29**	**[1.12, 1.48]**	**1.31**	**[1.08, 1.60]**
	Don’t Know	0.94	[0.66, 1.33]	0.63	[0.35, 1.14]
	Education				
	< High School	0.91	[0.70, 1.16]	1.06	[0.77, 1.47]
	High School/GED (ref)				
	College/Associates Degree	0.84	[0.66, 1.06]	0.76	[0.56, 1.02]
	Bachelor’s Degree	0.86	[0.67, 1.09]	**0.74**	**[0.56, 0.98]**
	Advanced Degree	0.82	[0.61, 1.10]	0.69	[0.46, 1.03]

*Note*. AOR = adjusted odds ratio; CI = confidence interval. NH = Non-Hispanic.

Results were based on weighted multinominal logistic regression weighted by PATH W7 cross-sectional weight for Wave 7 cohort data.

Boldface indicates statistically significant AORs (95% confidence interval does not include 1.0).

Among adults, nearly one-third were young adults aged 19–34 years (29.1%), and almost half were male (48.5%). The majority were non-Hispanic White (61.2%), and most had a Bachelor’s degree or higher (62.8%). Additionally, 53.6% were from families with an annual household income of $50,000 or higher.

Among adults who currently use tobacco, there were 183 distinct patterns of single and combined use across ten tobacco product types that were reported, together representing 24.7% of US adults overall; single, dual, and poly-use accounted for 17.2%, 5.5%, and 2.0%, respectively. As shown in Fig 2, overall, the most commonly used products were cigarettes, e-cigarettes, cigars, smokeless tobacco, and hookah (overall use). Among single users, cigarettes (9.8%), e-cigarettes (4.0%), cigars (1.7%), smokeless tobacco (1.0%), hookah (0.4%), and nicotine pouches (0.2%) were the top six products. The most frequently reported dual-use patterns included co-use of cigarettes and e-cigarettes (2.4%), cigarettes and cigars (1.2%), e-cigarettes and cigars (0.4%), e-cigarettes and hookah (0.2%), and e-cigarettes and nicotine pouches (0.1%). Among poly-users, the most common patterns include cigarette-e-cigarette-cigar (0.6%), cigarette-e-cigarette-hookah (0.1%) and other patterns less than 0.1%. Among the 370 nicotine pouch users specifically (S2 Fig), 20.3% were single users, 33.3% co-used with other products, including e-cigarettes (14.2%), cigarettes (7.7%), smokeless tobacco (4.7%), and cigars (2.1%). The majority of nicotine pouch users were poly-tobacco users (46.4%). Lastly, as shown in Table 2, among dual tobacco product users, compared with single-product users, males (AOR = 1.16, 95% CI = 1.02, 1.31), NH Black participants (AOR = 1.23, 95% CI = 1.06, 1.44), and individuals with lower annual household income (AOR = 1.29, 95% CI = 1.12, 1.48) had higher odds of dual-tobacco use, whereas older adults had lower odds compared with younger adults. Similar patterns were observed for poly-tobacco use: male (AOR = 1.87, 95% CI = 1.55, 2.27; NH Black participants (AOR = 1.48, 95% CI = 1.13, 1.94), and those with lower annual household income (AOR = 1.31, 95% CI = 1.08, 1.60) had higher odds of engaging in poly-tobacco use, while older adults had lower odds to do so.

**Fig 2:**
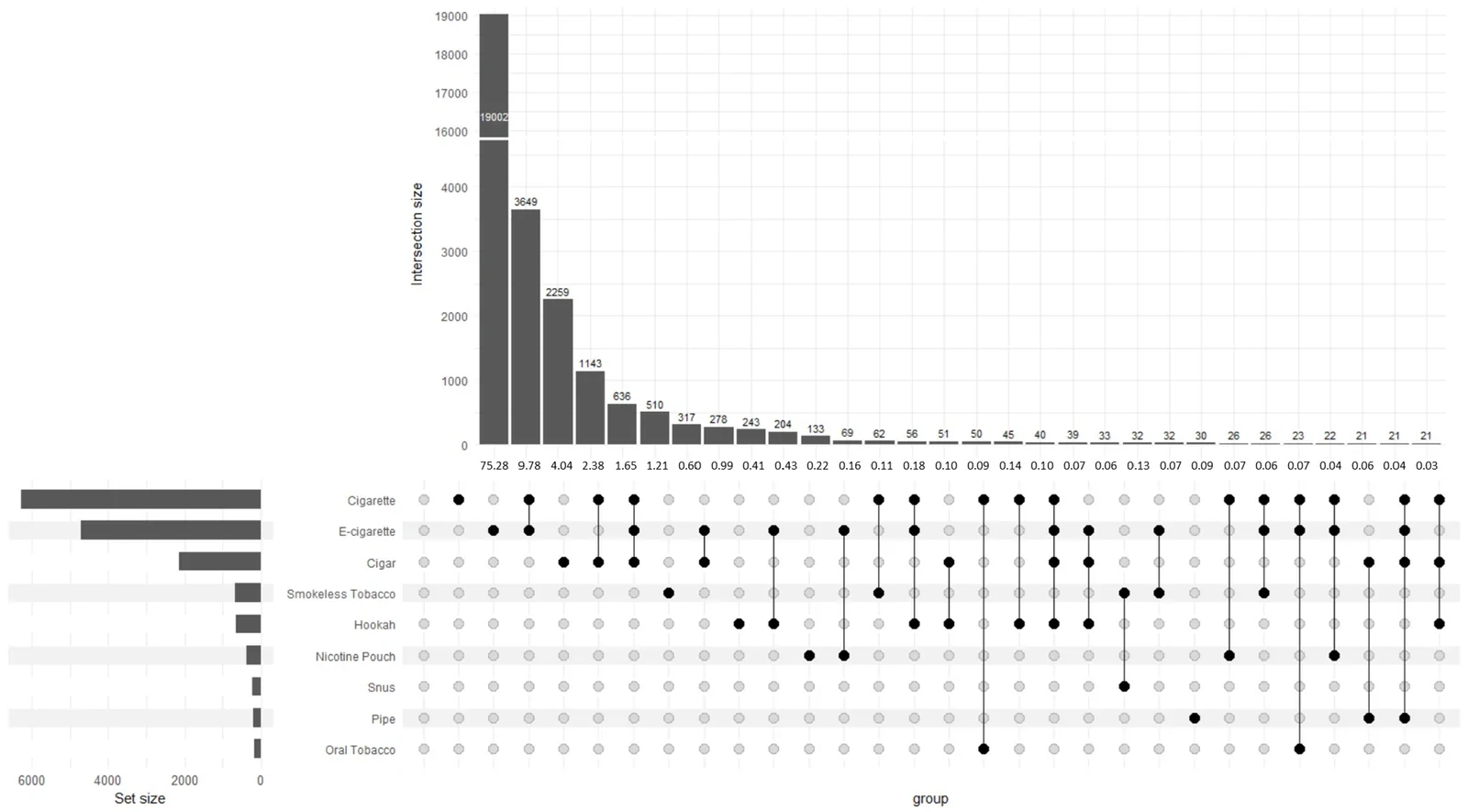
Upset plot of current tobacco use among adults (n = 29645). This plot, illustrating the intersection of binary current tobacco use, display: (1) a bar chart of intersection sizes (y-axis = frequency), and (2) a matrix of dots/lines indicating which products are included in each intersection (filled dot = included; empty = excluded), with side bars showing the sample size of each individual product used. Intersections were ordered by unweighted size, and weighted proportions were displayed below the corresponding bar. This figure displays up to 30 intersection patterns.

## Discussion

Using population-level PATH data collected in 2022–2023, we described the overlap in use across up to eleven tobacco products presenting the major tobacco and nicotine product types as well as emerging and novel products used in the U.S. Product selection was guided by the principal of surveillance and evaluation of products relevant to FDA tobacco regulatory science. Among youth, we identified more than 40 distinct product-use combinations. Most were exclusive single-product users, with the remainder reporting dual or poly use – consistent with the 2022 National Youth Tobacco Survey estimate that 3.5% of youth currently used two or more tobacco product types [20]. Among adults, we observed more than four times as many distinct product-use combinations as among youth, but the overall pattern was similar: most users were single-product users with a smaller proportion reporting dual or poly use. The prevalence of current dual use or poly-use is comparable to estimates from the National Health Interview Survey: 4% and 3.7%, respectively, in 2018 and 3.9% for the use of two or more tobacco products among U.S. adults in 2023 [29].

However, direct comparisons of dual- and poly-tobacco use with prior studies are often challenging because the range of tobacco products assessed may differ, and some studies may combine multiple products into a single composite category while others do not. For example, National Survey on Drug Use and Health (NSDUH) defines tobacco product categories to include cigarettes, e-cigarettes, smokeless tobacco (i.e., sniff, dip, chewing tobacco or snus), cigars (i.e., cigarillos, big cigars, and little cigars) and Pipe tobacco. Therefore, dual- and poly-tobacco use patterns identified in NSDUH and PATH data are not directly comparable unless the analyses are first harmonized to ensure consistent definitions of both the range of tobacco products included and the specific tobacco product categories. Our findings suggest that poly-tobacco use is associated with specific participant characteristics. In both youth and adults, males are more likely to be poly-product users compared to females. Dual and poly-use were generally less common among adults with higher education, higher annual household income, and older age. These patterns align with prior findings and potentially help identify priority subgroups for tobacco prevention and cessation efforts [21].

Ongoing surveillance of dual and poly-product use in national data is essential for tracking trends and identifying changing use patterns [30]. A key strength of this study is its use of recent, nationally representative US samples, providing comprehensive estimates of overlapping tobacco use among youth and adults. For each sample, we reported on overall prevalence and rank order use of up to eleven products, as well as provide information on single-, dual-, and poly-use combinations, enabling future work to examine specific patterns in greater depth, and provide information for potentially combining subcategories of use to allow sufficient sample size to overcome analytical challenges of study poly-use [31].

Our findings may also help inform tobacco prevention and cessation interventions. Among youth, the high prevalence of e-cigarette use highlights the importance of continuing to prioritize e-cigarette prevention efforts among tobacco naïve individuals. Among current tobacco users, the most commonly observed single-, dual-, and poly-tobacco use patterns involved e-cigarette use, suggesting that tobacco use interventions should also prioritize e-cigarette users. Among adults, prior studies suggest that some poly-tobacco users exhibit higher levels of nicotine dependence, indicating that these individuals may need additional cessation support. In addition, existing studies on cigarette cessation have largely focused on dual use of cigarettes and e-cigarettes, despite many users concurrently using other combustible and non-combustible products. Such information may help guide cessation interventions aimed at reducing nicotine dependence. Further, our results should encourage monitoring emerging products and their co-use with other products—for example, nicotine pouch use is increasing among adults and ranked as the sixth most commonly used product among youth and adults.

Among participants who reported nicotine pouch use, only 14.6% of youth and 18.6% of adults used nicotine pouches exclusively. The low prevalence of exclusive nicotine pouch use suggests that they are primarily used in combination with other tobacco products (mainly e-cigarettes) rather than as a standalone product [32,33]. Among youth, it would be critical to monitor whether youth initiate nicotine pouch use as their first tobacco product, as nicotine pouches are less restricted by location of use and may be perceived as less harmful than e-cigarettes. Nicotine pouch use may also become integrated into broader patterns of experimentation of multiple tobacco products. Among adults, nicotine pouches may be supplementing rather than replacing other tobacco products, particularly cigarettes and e-cigarettes, although longitudinal studies are required to determine whether these apparent use patterns are stable. These findings highlight the importance of surveillance and regulation of emerging nicotine products within the broader context of poly-tobacco use rather than evaluating nicotine pouch use in isolation.

This study has several limitations. First, tobacco use was self-reported and may be subject to recall error and underreporting. Second, tobacco use patterns continue to evolve; therefore, our findings reflect only the study period examined. This highlights the importance of continued collection and evaluation of tobacco surveillance data. In addition, the findings are based on the U.S. non-institutionalized population and may not generalize to other countries, or excluded populations (e.g., individuals experiencing homelessness or military populations). Third, patterns of tobacco use were defined based on frequency of use in the past 30 days. Although alternative measurements (e.g., intensity of poly-tobacco use; biomarkers of exposure) may provide a more nuanced characterization of tobacco use behavior, their implementation remains methodologically and analytically challenging [31]. As a result, the implementation and examination of harmonized measures of tobacco use remains limited and warrants further methodological development. Fourth, our analysis was primarily descriptive and focused on sociodemographic correlates, so causal inferences cannot be made. Longitudinal studies are needed to examine transitions among single, dual, and poly-tobacco use and to assess determinants and health consequences of these changes [34–36].

In conclusion, although most youth and adults use a single tobacco product, a substantial proportion engage in dual or poly use, often involving multiple product combinations and disproportionately affecting males, non-Hispanic adults, and those with lower socioeconomic status. Tobacco surveillance and research should routinely assess a broad range of products, including emerging ones. These findings support policies that address multiple-product use and intervention efforts that target high-risk subgroups and explicitly address co-use rather than focusing on single products alone.

## Supporting information

S1 Fig Upset plot of current tobacco use among youth nicotine pouch users (n = 28). This figure, illustrating the intersection of binary current tobacco use, displays: display: (1) a bar chart of intersection sizes (y-axis = frequency), and (2) a matrix of dots/lines indicating which products are included in each intersection (filled dot = included; empty = excluded), with side bars showing the sample size of each individual product used.
(DOCX)

S2 Fig Upset plot of current tobacco use among adult nicotine pouch users (n = 370). This figure, illustrating the intersection of binary current tobacco use, displays: (1) a bar chart of intersection sizes (y-axis = frequency), and (2) a matrix of dots/lines indicating which products are included in each intersection (filled dot = included; empty = excluded), with side bars showing the sample size of each individual product used. This figure displays up to 30 intersection patterns.
(DOCX)

S1 Table Description of study variables among youth. (DOCX)

S2 Table Description of study variables among adults. (DOCX)
