# Supplementary material for: Patterns of tobacco product single and co-use – Findings from the Population Assessment of Tobacco and Health Study (PATH) wave 7 data
Source: PLoS One. 2026 Nov 3;21(11):e0360653. doi: 10.1371/journal.pone.0360653 (PMC13730317; doi:10.1371/journal.pone.0360653)
Supplement: Supplementary file 4 [file pone.0360653.s004.docx]

|  | **PATH Variable** | **Label** | **Response Category** | **Recoded Response Category** |
| --- | --- | --- | --- | --- |
| Age Group | R07R_A_AGECAT6 | Age (in years) at the time of interview. | 1 = 18 to 24 years old;  2 = 25 to 34 years old;  3 = 35 to 44 years old;  4 = 45 to 54 years old;  5 = 55 to 64 years old;  6 = 65 years old or older | 1 = 18 to 24 years old;  2 = 25 to 34 years old;  3 = 35 to 44 years old;  4 = 45 to 54 years old;  5 = 55 to 64 years old;  6 = 65 years old or older |
| Sex | R07R_A_SEX_IMP | PATH imputed gender | 1 = Male;  2 = Female | 0 = Female;  1 = Male |
| Race/Ethnicity | R07R_A_RACECAT3 | Race (3 levels) | 1 = White alone;  2 = Black alone;  3 = Other | (recoded into four binary variables)  Hispanic (0 = No; 1= Yes);  NH White (0 = No; 1= Yes);  NH Black (0 = No; 1= Yes);  Other (0 = No; 1= Yes); |
|  | R07R_A_HISP_IMP | PATH imputed Hispanic origin | 1 = Hispanic;  2 = Not Hispanic |  |
| Education | R07R_A_AM0018_V2 | What is the highest grade or level of school you completed? (5 levels) | 1 = Less than High School  2 = GED  3 = High school graduate  4 = Some college (no degree) or associates degree  5 = Bachelor's degree or advanced degree | 1 = Less than high school  2 = GED  3 = High school graduate  4 = Some college (no degree) or associates degree  5 = Bachelor's degree or advanced degree |
| Poverty Status | R07R_A_AM0030  R07_AM0031 | Which of the following categories best describes your total household income in the past 12 months?  Is your total household income during the past 12 months above or below $50,000? | 1 = Less than $10,000  2 = $10,000 to $24,999  3 = $25,000 to $49,999  4 = $50,000 to $99,999  5 = $100,000 or more  1 = Above $50,000  2 = Below $5T0,000 | 0 = $50,000 or more;  1 = less than $10,000 - $49,999;  2 = Don’t Know |
| **Tobacco Use (PATH derived variables)** | | | | |
| Cigarette | R07R_A_P30D_CIGS | Have smoked a cigarette within the past 30 days. | 1 = Yes; 2 = No | 0 = No; 1= Yes |
| E-cigarette^a^ | R07R_A_P30D_EPRODS | Have used an electronic nicotine product within the past 30 days,  including: e-cigarettes, pod devices, vape pens, tank systems, mods, e-cigars, e-pipes, e-hookahs, and hookah  pens. | 1 = Yes; 2 = No | 0 = No; 1= Yes |
| Cigar^b^ | R07R_A_P30D_CIGAR: | Have smoked a cigar product (traditional cigar, cigarillo, filtered cigar) within the past 30 days. May include some  respondents who smoked cigars only as blunts. | 1 = Yes; 2 = No | 0 = No; 1= Yes |
| Pipe | R07R_A_P30D_PIPE | Have smoked a pipe within the past 30 days. | 1 = Yes; 2 = No | 0 = No; 1= Yes |
| Hookah | R07R_A_P30D_HOOK | Have smoked a hookah within the past 30 days. | 1 = Yes; 2 = No | 0 = No; 1= Yes |
| Snus | R07R_A_P30D_SNUS | Have used snus within the past 30 days. | 1 = Yes; 2 = No | 0 = No; 1= Yes |
| Smokeless Tobacco^c^ | R07R_A_P30D_SMKLS | Have used smokeless tobacco within the past 30 days. | 1 = Yes; 2 = No | 0 = No; 1= Yes |
| IQOS | R07R_A_P30D_IQOS: | Have used IQOS within the past 30 days. | 1 = Yes; 2 = No | 0 = No; 1= Yes |
| Nicotine Pouch | R07R_A_P30D_NP | Have used nicotine pouches within the past 30 days. | 1 = Yes; 2 = No | 0 = No; 1= Yes |
| Oral Tobacco^d^ | R07R_A_P30D_OT | Have have used oral tobacco within the past 30 days. | 1 = Yes; 2 = No | 0 = No; 1= Yes |
|  |  |  |  |  |

^a^ E-cigarettes, or Electronic nicotine products, include e-cigarettes, pod devices, vape pens, tank systems, mods, e-cigars, e-pipes, e-hookahs, and hookah pens.

^b^ Cigars include traditional cigar, cigarillo, or filtered cigar.

^c^ Smokeless Tobacco include dip, spit, moist snuff, pouches, or chewing tobacco

^d^ Oral Tobacco: This item concerns oral nicotine products that one put in mouth, other than nicotine pouches. This includes lozenges, discs, tablets, gum, toothpicks, dissolvable tobacco, and related products. Common brands include Velo Nicotine Lozenges, Verve Nicotine Discs, Rogue Nicotine Tablets, Lucy Nicotine Gum, Pixotine Nicotine Toothpicks, and Stonewall Dissolvable Tobacco. This item also excludes nicotine replacement therapy products.
